# The Post-transplant Lymphoproliferative Disorders—Metagenomic Shotgun Microbial Sequencing (PTLD-MSMS) Study Methods and Protocol

**DOI:** 10.1097/TXD.0000000000001723

**Published:** 2024-10-28

**Authors:** Vikas R. Dharnidharka, Kristine M. Wylie, Todd N. Wylie, Marianna B. Ruzinova, Charles W. Goss, Gregory A. Storch, Neha Mehta-Shah, Derek Byers, Leslie Walther, Lujain Jaza, Hongjie Gu, Mansi Agarwal, Michael Green, Erika Moore, Steven H. Swerdlow, Fernanda Silveira, Lianna J. Marks, Dita Gratzinger, Adam Bagg, Soi Cheng Law, Maher Gandhi

**Affiliations:** 1 Washington University School of Medicine, St. Louis, MO.; 2 UPMC Children’s Hospital of Pittsburgh, Pittsburgh, PA.; 3 University of Pittsburgh School of Medicine, Pittsburgh.; 4 Stanford University School of Medicine, Palo Alto, CA.; 5 University of Pennsylvania, Philadelphia, PA.; 6 Mater Research, University of Queensland, Woolloongabba.

## Abstract

Post-transplant lymphoproliferative disorders (PTLDs) remain a feared complication of transplantation, with significant morbidity and mortality. The oncogenic Epstein-Barr virus (EBV) is a key pathogenic driver in 50%–80% of cases. Numerous prognostic indices, comprising multiple clinical, epidemiological and tumor characteristics, including EBV tumor positivity, do not consistently associate with worse patient survival, suggesting a potential role for EBV genome variants in determining outcome. However, the precision medicine tools for determining if a viral genome variant is pathogenic are very limited compared with human genome variants. Further, targeted studies have not implicated a specific viral etiological agent in EBV-negative PTLD. Using novel cutting-edge technologies, we are extracting viral nucleic acids from formalin-fixed, paraffin-embedded archived, or frozen PTLD tissues or plasma, to test for all vertebrate viruses simultaneously in an unbiased fashion, using metagenomic shotgun sequencing (MSS). We are collecting such samples from multiple transplant centers to address the following specific aims and close the following knowledge gaps: (1) Validate our novel observation that PTLD tissue positivity by MSS for anellovirus (and confirmed by PCR) serves as a biomarker for higher transplant recipient mortality after the diagnosis of PTLD; (2) determine the role of other oncogenic viruses in EBV-negative PTLD by unbiased MSS of multiple viral groupings, confirmed by other techniques; and (3) develop the necessary computational, algorithmic and software analytic tools required to determine association of EBV genome variants with worse presentations or outcomes in PTLD. Study completion will contribute to better patient care and may provide avenues for novel therapies.

## Graphical abstract

**Figure:**
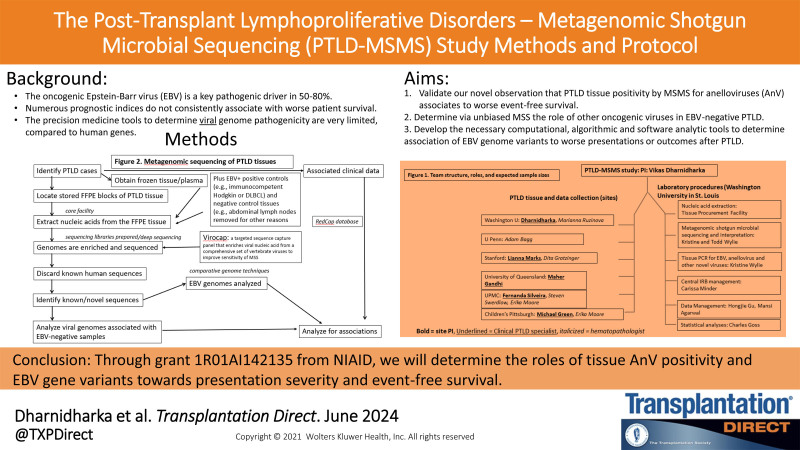

## INTRODUCTION

Post-transplant lymphoproliferative disorders (PTLDs) remain a serious and often devastating complication of solid organ transplantation and, less commonly, hematopoietic stem cell transplantation, since first being reported in 1969.^1-4^ PTLDs, also referred to as post-transplant lymphoproliferative disease, is not one condition but a heterogeneous spectrum of disorders. PTLDs are characterized by abnormal proliferation of lymphoid immune cells, in the context of impaired immune surveillance from the extrinsic immunosuppression that is needed to prevent acute rejection of the transplanted organ.^5-7^ Several editions of WHO classifications include a spectrum of post-transplant lymphoproliferative processes from hyperplasia to frank lymphoma.^8,9^

PTLDs occur relatively rarely, thus restricting any large multicenter studies or prospective trials. The incidence density for PTLD ranges from 1.58 per 1000 person-years (kidney) to 2.24 (heart), 2.44 (liver), and 5.72 for lung.^10^ In the French PTLD registry, cumulative incidence was 1% by 5 y and 2.1% by 10 y.^11^

Around 50%–80% of cases are strongly associated to the oncogenic Epstein-Barr virus (EBV), by epidemiological, tumor staining, and in vitro mechanistic criteria, such that EBV is believed to be a key pathogenic driver in EBV-positive PTLDs. However, several knowledge gaps in pathogenesis still persist, for example, why the timing, location, and severity of PTLD is so variable, why treatments have such variable outcomes, or what role EBV genome variants play in lymphoid cell transformation. Several groups have developed prognostic indices to predict patient survival at time of PTLD development.^1^ Many of these indices were derived from similar indices used in Hodgkin lymphoma and non-Hodgkin lymphoma, but more recent indices are specific to PTLD.^12^ No indices or individual criteria are consistently able to predict worse post-PTLD outcomes of earlier patient death or earlier death-censored allograft failure.

EBV has a genome of 172 k base pairs and 85 genes, with considerable variation in gene sequence between strains^13,14^ that may affect its virulence. But the recognized variants in these genes have not been studied for association to more severe presentation or worse post-PTLD outcomes. In contrast, human genome variants have been studied, with no specific pattern emerging.^15-17^ No virus etiology, or any other microbial etiology, has as yet been detected in those PTLDs, which are EBV-negative by in situ hybridization or immunohistochemistry. Other studies have suggested that EBV-negative PTLDs have a different pathogenesis,^18^ perhaps related more to dysregulated lymphoid proliferation.^19^

Using novel recently developed technologies to interrogate PTLD tissues for new biomarkers and to advance our understanding of its cause and severity, we were able to (A) successfully extract DNA from formalin-fixed, paraffin-embedded archived PTLD tissues from 1991 to 2015, up to 25 y post-fixation; and (B) successfully sequence multiple DNA viruses simultaneously, using metagenomic shotgun sequencing (MSS) and ViroCap.^20^ Upon abstracting detailed, well-annotated clinical and pathological data on our center’s cases, we discovered that tissue positivity for anellovirus by MSS (any reads) associated with higher patient death within 5 y of diagnosis (*P* = 0.03 in contingency analyses, *P* = 0.02 for confirmatory quantitative PCR in time-to-event Cox regression), a novel finding that may represent over-immunosuppression as anelloviruses have no known pathological role.^21^ We have been funded by the National Institutes of Allergy and Immunological Diseases branch of the National Institutes of Health (grant number AI-R01-142135) to validate our findings and perform additional studies in the multicenter PTLD-MSMS study with the following aims:

1. Validate our novel observation that PTLD tissue positivity by MSS for anellovirus serves as a biomarker for higher transplant recipient mortality after the diagnosis of PTLD.
2. Determine the role of other oncogenic DNA viruses in EBV-negative PTLD.
3. Develop the necessary computational, algorithmic, and software analytic tools required to then determine association of EBV genome variants, in EBV-positive PTLD, to close the stated knowledge gaps.

## MATERIALS AND METHODS

We will procure both retrospectively collected PTLD tissues and prospectively collected PTLD tissues/plasma (proposed n = 630) from several locations: (1) cases at Washington University that occurred after 2015; (2) USA and Australian sites with large transplant programs and existing PTLD collections (Universities of Pittsburgh and Queensland, Stanford University). We will (a) acquire tissue scrolls from the PTLD samples (plus plasma if prospective) and transport to Washington University; (b) extract the microbial and human DNA and RNA; (c) perform MSS for virus detection, confirmed by tissue PCR; and (d) validate our novel anellovirus associations by enriching the dataset to include data from multiple sites. Our multidisciplinary, international team of specialists in transplantation, hematopathology, infectious diseases, oncology, genomics, and statistics will use the MSS and other technologies and this larger multicenter sample cohort to achieve the stated aims.

## APPROACH

### Team Structure and PTLD Identification

**FIGURE 1.**
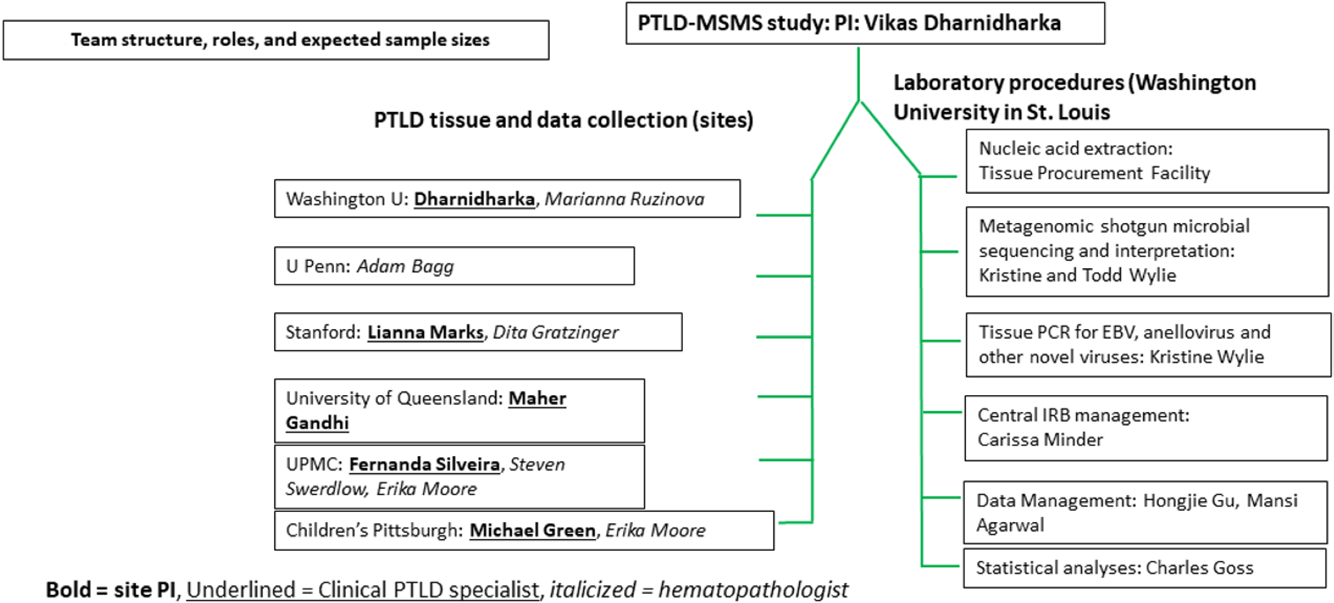
The team structure, roles, and procedures in the PTLD-MSMS study. PTLD-MSMS, post-transplant lymphoproliferative disorders metagenomic shotgun microbial sequencing.

We will conduct a 5-y multicenter study across the sites listed in Figure 1. Each site is headed by a clinical PTLD specialist and a hematopathologist with an interest in PTLD. Each site was selected based on the following characteristics: (a) the site is a high-volume transplant program; (b) it has a large PTLD tissue collection; and (c) at least one of the site investigators is a senior investigator in the PTLD field.

The tissue samples collected at each site will be either retrospective (already archived; most samples; sent to central site in the first 2 y) or prospective (some samples, collected in the first 3 y of the 5-y study). To capture prospective cases across each of the large institutions, we will send a blast email to the various transplant teams at each institution once the study starts, asking them to notify the site PI by email or phone in advance if they have a biopsy for potential PTLD. We will also send a refresher email quarterly. Finally, we will post flyers in the various transplant clinics and at nursing stations of inpatient transplant floors.

### Tissue Sample Identification, Tissue Scrolls, and Plasma

We will first identify through a search of our electronic medical records all formalin-fixed paraffin-embedded (FFPE) tissue blocks from PTLD cases available in the tissue archives of the study sites. Tissue specimens of adequate quantity (as evaluated by the site hematopathologist) will be selected for nucleic acid extraction. Ten tissue scrolls (aka shavings; curls) at 10 µm will be harvested from the FFPE blocks by the site pathology (histology) technologist. Scrolls will be stored in an Eppendorf tube and batch shipped at room temperature to Washington University. Prospective fresh tissue samples will be snap frozen on aluminum foil or in a plastic OCT mold. With each prospective tissue sample, we will also obtain EDTA plasma samples from the subject, both after informed consent, and both shipped overnight to Washington University. Once received at Washington University, the frozen tissue will be embedded in OCT to enable subsequent histological evaluation and/or molecular specimen processing.

### Total Nucleic Acid Extraction (Tissue Procurement Facility, Washington University)

Scrolls from FFPE and snap-frozen tissue will be submitted for DNA extraction following a process of deparaffinization (FFPE only), enzymatic digestion, and silica column purification using the QIAamp DNA micro Kit (Qiagen, Venlo, The Netherlands). Total RNA will be isolated from frozen tissue scrolls using TRIzol reagent (Ambion) followed by RNA clean-up and DNase treatment using the RNeasy Micro kit (Qiagen). Plasma samples will be processed for total nucleic acid using the Maxwell RSC Viral Total Nucleic Acid Kit (Promega, Madison, Wisconsin). The concentration of nucleic acid is determined via the Qubit 2.0 Fluorometer (Qubit dsDNA HS/BR Assay Kit or Qubit dsRNA HS/BR Assay Kit), the purity of the preparations will be assessed via NanoDrop 2000 (A260/A280) and the integrity determined via Agilent Tapestation 4200 (RIN/DIN). Extracted nucleic acids will be stored at the facility at −80°C until analyzed.

### Metagenomic Shotgun Microbial Sequencing (McDonnell Genome Institute, Washington University)

**FIGURE 2.**
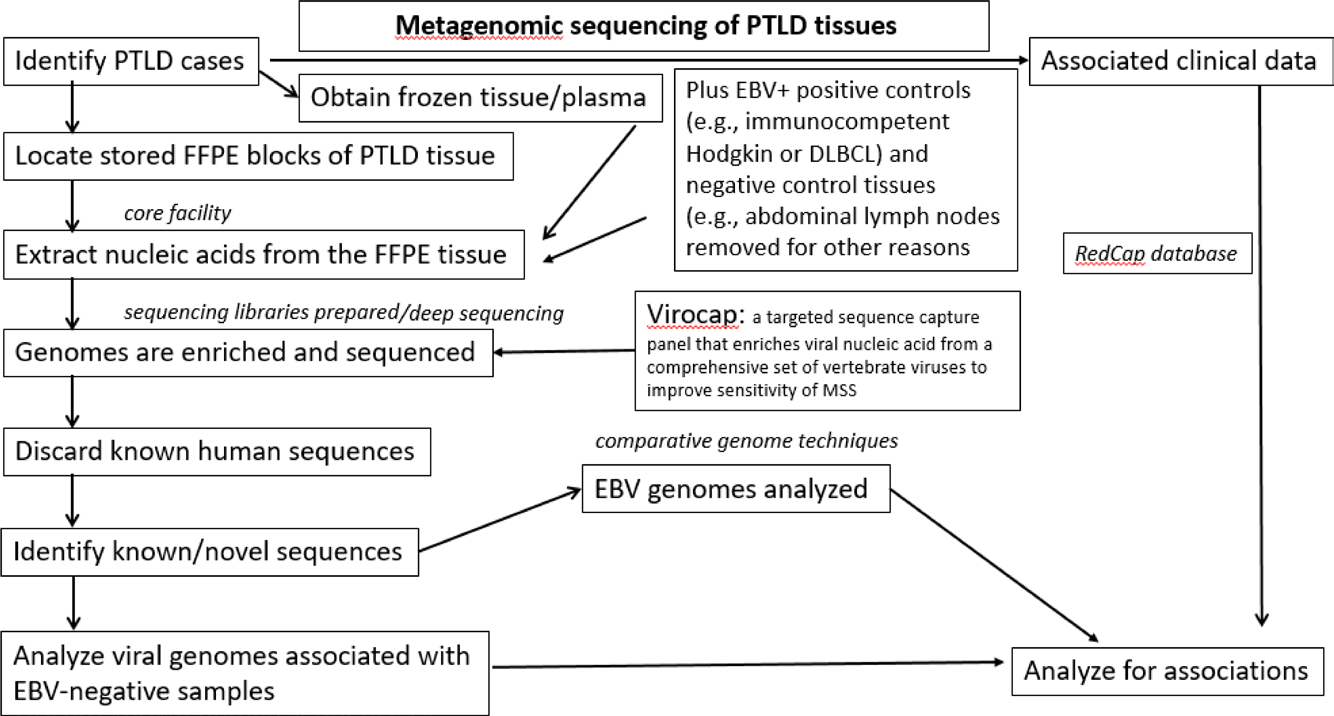
The procedures involved in the nucleic acid extractions and metagenomic shotgun sequencing of the PTLD tissues. PTLD, post-transplant lymphoproliferative disorder.

We will use metagenomic shotgun sequencing (1) to evaluate EBV genomes to identify variants associated with PTLD outcomes and (2) to determine whether other viruses besides EBV are associated with PTLD. To do this, we will use ViroCap, which enhances sensitivity of virus detection by comprehensively targeting and enriching complete genomes from all known vertebrate viruses.^20^ The genomes targeted include DNA viruses (herpesviruses, polyomaviruses, anelloviruses, etc.) and RNA viruses (influenzaviruses, hepatitis C viruses, pegiviruses, etc.). We will generate dual-indexed sequencing libraries from the extracted nucleic acids using the KAPA HyperPrep Kit with Library Amplification (Roche, Indianapolis, Indiana) or for input quantities <50 ng the xGen cfDNA & FFPE DNA Library Kit v2 MC (Integrated DNA Technologies, Coralville, Iowa). As shown in Figure 2, we will pool libraries and mix them with the ViroCap targeted sequence capture probes (custom designed by our group and synthesized by Roche Nimblegen, Madison, WI) according to the manufacturer’s instructions. We will sequence the enriched viral nucleic acids using the Illumina NovaSeq platform (llumina, San Diego, CA). We will analyze sequences using the ViroMatch pipeline,^22^ which uses the Burroughs Wheeler Alignment tool BWA mem for nucleotide sequence alignments^23^ and DIAMOND for translated alignments.^24^ Sequences will be manually reviewed to verify classification of herpesvirus and polyomavirus sequences. Any positive reads, as determined by the ViroMatch Pipeline, will be considered as MSS positive for a virus (binary Yes/No outcome, to be confirmed by tissue PCR for that virus). For samples that are positive for EBV sequences, sequences will be aligned to canonical EBV-1 and EBV-2 reference genomes (NCBI Reference Sequence: NC_007605.1 and NC_009334.1). The depth and breadth of read coverage and sequence alignments will be reviewed to determine distinct EBV types. Variants in the EBV-1 genome will be identified using VarScan, a platform-independent software tool developed at the McDonnell Genome Institute at Washington University to detect variants in MSS data.^25^ Variants and coverage are manually reviewed using Tablet, a high-performance graphical viewer for metagenomic sequence assemblies and alignments.^26^ Multiple positive and negative control tissues will be used (Figure 2).

### Polymerase Chain Reaction

Tissue specimens that are positive for EBV and/or anellovirus by next generation sequencing will be subject to quantitative PCR confirmation. We will identify or develop assays as needed for other viruses. To quantify EBV in the specimens, a quantitative TaqMan real-time PCR assay developed in our laboratory will be employed. The primers and TaqMan probe used are contained within an amplicon originally described by Wandinger et al.^27^ Primer Express was used to design a set of TaqMan primers and a TaqMan MGB probe based on this amplicon. The sequences detected by these primers and probe are found within the EBV EBNA-1 gene: forward primer EBV-F: 5′ GGT-AGT-AAG-ACC-TCC-CTT-TAC-AAC-CT 3′, reverse primer EBV-R: 5′ TGT-AAG-ACG-ACA-TTG-TGG-AAT-AGC-A 3′, probe EBV-MGB: 5′ 6FAM-CGA-GGA-ACT-GCC-C-MGBNFQ 3′. The PCR reaction will be performed in a final reaction volume of 25 µL using ABI TaqMan Universal PCR master mix and including 5 µL of specimen nucleic acid extract. The final concentrations of each primer and probe are 0.9 and 0.25 µM, respectively. The reaction is run in an Applied Biosystems 7500 Real-Time PCR System instrument with a program of 50°C for 1 min, 95°C for 10 min, 40 cycles of 95°C for 15 s, and 60°C for 1 min. A set of standards, consisting of a series of six 10-fold dilutions of a plasmid containing the cloned EBV target, is included in each run. To quantify the amount of human DNA in each specimen, the Applied Biosystems Quantifiler Human DNA Quantification Kit (Cat No. 343895; Foster City, CA) is run according to kit instructions. Results are expressed as copy number of EBV per µg of human DNA.

To quantify the TTV (alphatorquevirus) species of anellovirus, TaqMan quantitative real-time PCR will be performed to detect alphatorquevirus in extracted samples, using an amplicon that was previously described.^28^ The alphatorquevirus assay targets a highly conserved segment of the viral untranslated region. Each alphatorquevirus reaction is performed in 25 μL total volume, including 5 μL extracted specimen, ABI TaqMan Universal PCR Master Mix (Applied Biosystems), 0.9 μM forward primer (5′ TGCCGAAGGTGAGTTTACACA 3′), 0.9 μM reverse primer (5′ TTCAGAGCCTTGCCCATAGC 3′), and 0.25 µM probe (5′ 6fam-CCCGAATTGCCCCTTGAC-MGBNFQ 3′). Cycling is carried out on the ABI 7500 instrument with the following conditions: 50°C for 2 min and 95°C for 10 min, followed by 45 cycles of 95°C for 15 s and 60°C for 1 min. The quantitation standard consists of a synthesized 144 bp region from the conserved, untranslated alphatorquevirus genome, which contains the target for PCR, inserted into the pUC57 plasmid. Tenfold dilutions of the plasmid are used for quantitative standards. Results are expressed as copy number of TTV per µg of human DNA.

### Pathology and Clinical Covariates

From the local pathology electronic medical record, the following data elements have been extracted by the data collectors at each site into the central study database: (1) Final clinical diagnosis/pathological classification based on the 4th edition WHO classification that was in place the time of our database construction (PTLD, polymorphic/monomorphic, further classification of lymphoma subtype in cases of monomorphic PTLD); (2) lineage determination markers (B cell, T/NK cell, plasma cell), EBV tumor status (EBER1 in situ hybridization or LMP1/EBNA immunohistochemistry); and (3) ancillary test results including data from flow cytometry, molecular, and cytogenetic analysis when available. The local site hematopathologist will be available to answer queries from the site data collector or the central research coordinator during quality control checks.

Clinical data elements will be extracted by each site’s data collector from the site’s electronic medical record into the central study REDCap database: (1) Recipient demographics (age at transplant, sex, race, age at PTLD); (2) donor demographics (age at donation, sex, race); (3) recipient sero-status (EBV and Cytomegalovirus); (4) donor sero-status (EBV, Cytomegalovirus); (5) recipient transplant (type of organ/hematopoietic transplant, date of and age at transplant, induction/maintenance immunosuppression); (6) donor type (related/unrelated, deceased or living, standard/extended/cardiac death, HLA matching to recipient); (7) PTLD date of diagnosis; (8) location(s) of PTLD; (9) treatment immunosuppression after PTLD diagnosis: fold immunosuppression reduction/discontinuation of calcineurin inhibitors, antimetabolites, steroids; (10) other systemic PTLD therapy (chemotherapy, rituximab, intravenous immunoglobulin, antiviral drugs); (11) PTLD outcomes (complete versus partial remission, recurrence); and (12) patient and allograft survival after PTLD (out to 5 y in retrospective cases, out to 2 y in prospective cases). The timeline of the various study events is shown in Table 1.

**TABLE 1.**
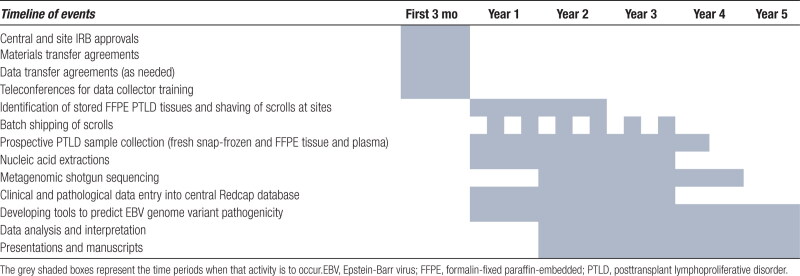
Timeline of the planned study events

### Classification of Difficult Cases

A small subset of PTLD cases are difficult to classify, sometimes due to small tissue size, and thus may be difficult to place in existing categories. These cases are placed in the “Other” category in central study REDCap database and may be reviewed by our Pathology review panel that includes all of the participating hematopathologists, to see if a consensus diagnosis for the case may be reached. One such meeting has already occurred virtually, and additional meetings may take place as needed.

### Role of Other DNA Viruses in EBV-Negative PTLD

With 27/69 (39%) of our PTLD tissues being EBV negative, we estimate that 246 PTLD tissue samples (39%) in the larger cohort will be EBV negative. Our samples so far suggest that a specific DNA viral genus other than EBV is not dominant in EBV-negative PTLDs. However, we recognize that our previously published cohort was too small to provide definite conclusions. The power of the larger cohort of 246 EBV-negative PTLDs, compared with 384 EBV-positive PTLDs, will allow us to perform contingency analyses to determine which of the 15 DNA viral taxa are positive by MSS in a significantly greater proportion in the EBV-negative cases, to a high degree of statistical significance. If a specific virus or viral group (eg, polyomaviruses) is found, we will confirm this result by other techniques such as tissue PCR. If no viral genus is positive by MSS in a significantly greater proportion of the EBV-negative cases, we will have sufficient power to make that conclusion with a high degree of certainty.

### Developing the Algorithms and Computational Tools to Identify Genome Variants Associated With Outcome

We will have an efficient analysis pipeline coupled with a larger data set that will provide more power to detect common gene variation and to associate specific genetic variants with clinical outcomes and WHO histological classifications. We will use Python software^29^ to generate the pipeline, which will integrate robust software tools, including BWA mem^23^ and Varscan.^25^ Databases will be created and referenced for EBV genomes and annotation, genome features (repeat regions), and previously characterized mutations associated with viral pathogenicity.

This pipeline will be applied to the analysis of the EBV-positive samples in this study. Using in part some commercially available products, we will create an automated computational pipeline, which we will make publicly and freely available to other investigators, that will take unaligned, Illumina sequencing data, align to EBV reference genomes, report coverage statistics, identify variant sequences, determine whether the variants are in coding regions, identify which genes contain the variants, determine whether the changes lead to synonymous or nonsynonymous changes, and generate reports that identify a discrete list of regions suitable for manual review and/or confirmation by PCR assay. These reports will flag variants that will require particularly careful review by an analyst, such as variants that occur in or near repetitive genomic regions. The report will also flag variants that have previously been reported in the literature and have functional characterization.

### Overall Study Approach Considerations

#### Regulatory Management

At Washington University, we have conducted our single-center retrospective study under Washington University institutional review board approval and a waiver of consent. The proposed study is being conducted with Washington University serving as the single institutional review board, as required by the NIH. We have been granted a waiver of consent for retrospectively stored PTLD tissue samples use in the proposed study. For prospective tissue and plasma samples, we obtain informed consent before any the samples are used for this research. As needed, we completed a Materials Transfer agreement or Data Transfer agreement with the sites.

#### Data Management and Quality Control of Data

We have already constructed a secure, study database using the Research Electronic Data Capture (REDCap) system. We have developed and refined the data-collection instruments over the past 3 y, as we have conducted our single-center study. The database links each set of forms through a common ID number. The different instruments within the database incorporate demographic, peritransplant, initial immunosuppression, PTLD date/location/pathological diagnosis, associated clinical virology data, initial intervention post-PTLD, responses, outcomes, and genomic data. Supporting instructions, defined field formats and limiters are used to ensure consistent, high quality data. Automated data queries are generated if an entry is outside of the preset boundaries or an illogical value. All data collectors at the various sites will undergo and pass a mandatory training webinar conducted by the Washington University central site research coordinator, who is already familiar with the database. The clinical PTLD specialist at each of the sites will be responsible for randomly auditing 25% of the chart entries. The study research coordinator will review each form received and generate any additional manual queries. Overall data management and quality controls will be under the supervision of our data management team.

### Statistical Analyses

Outcomes (worse presentation severity such as disseminated or Burkitt or patient death or death-censored graft failure/repeat PTLD) and explanatory variables (demographic characteristics, type of organ transplant, timing of PTLD, tissue EBV or anellovirus positivity by MSS or PCR, PTLD treatment and imaging, PTLD tumor sample characteristics and EBV genetic variants) will be summarized using descriptive statistics. As in our prior single-center study, EBV genomic variant analysis will initially focus on those EBV genes most associated with oncogenesis or viral latency patterns and on those nonsynonymous nucleotide changes that lead to a change in the coded amino acid, but the much larger sample here will allow us to explore associations to variants in all other EBV genes. We will report categorical variables as N (%) and continuous variables will be reported as mean ± SD or median (IQR), as appropriate. Explanatory variables will be summarized both overall and stratified by the different outcome groups. Time to death and death-censored graft failure/repeat PTLD outcomes (within 5 y of PTLD diagnoses) will be analyzed using Kaplan-Meier survival analysis and Cox regression. To evaluate associations between categorical variables and time to event outcomes, we will use log-rank tests and plot Kaplan-Meier survival to illustrate survival patterns over the course of the study. Variables that demonstrate some evidence of an association (eg, *P* < 0.10) and those variables thought to be a priori biologically important predictors will be included in a multivariable Cox regression model to obtain adjusted hazard ratios and 95% confidence intervals. *P* values <0.05 will be considered as significant. All analyses will be carried out using SAS version 9.4 (Cary, NC).

To efficiently carry out statistical comparisons of viral genetic data, options will be available to create tables of coverage, nucleotide variants, and amino acid variants for gene sets from a set of sequenced samples for comparison. These tables will be uploaded into databases or statistical software for further analysis. Files containing nucleotide and amino acid sequences of individual genes will also be created to be available for further downstream, comparative analyses.

### Power Analyses

#### Aim 1

The goal of this aim is to compare mortality among subjects whose PTLD tissue is positive as compared with negative for the anellovirus (AnV). Prior data from 69 tissue samples suggest that we will have an approximately equal number of tissue samples that are positive and negative for this virus. Those same data found a mortality hazard rate ratio of 2.0 in the positive group as compared with the negative group, with the actual hazard ratios in the two groups being 0.8 in the AnV-positive group and 0.4 in the AnV-negative group over a 5-y period. With these data, only 24 subjects per group are required to achieve a power of 0.9 for a two-sided log rank test comparing mortality curves under the assumption of an exponential distribution. If the hazard rate ratios are assumed to be 0.8 and 0.6, 65 per group are required to achieve a power of 0.9, while hazard rates of 0.8 and 0.7 require 128 per group for a power of 0.9. These data indicate that total target sample size of 630 tissue samples will be more than sufficient to evaluate the effects of interest for this aim. There are two reasons for studying the large number of samples. First, we will be interested in evaluating the mortality effect of AnV status in subgroups such as those with T-cell PTLDs or PTLD location in liver or bone marrow (each of which is a small subgroup of 6 or less within the 69, but with much higher mortality than the others). Second, the large sample size is required to achieve the narrow confidence bounds that are desired in the second aim.

#### Aim 2

The central hypothesis of this aim is that the prevalence of particular viral genus groupings in tissue from PTLD patients will be similar in EBV-neg tissue as compared with EBV-pos tissue. Because this is an equivalence hypothesis, our sample size considerations will be based on 95% confidence bounds. Specifically, we will assume that a particular viral grouping has the same prevalence in EBV+ and EBV− tissue. Based on the target sample size, we will then compute the 95% confidence bounds that quantify the true possible difference in viral grouping prevalence by EBV status. That is, if *P*+ is the prevalence of a viral grouping in EBV positive tissue and *P*− is the prevalence of that grouping in EBV− tissue, we will compute the confidence bounds on the difference (*P*+ - *P*−) under the assumption that the true values of *P*+ and *P*− are the same. Since prior data suggest that, depending on the grouping, viral prevalence will range from 5% (eg, simplexvirus and parvoviruses) to 50% (eg, anellovirus and roseolovirus), our computations will be performed under the assumption that viral grouping is present in 5%, 10%, 20%, 30%, 40%, and 50% of EBV+ and EBV− tissue.

The sample size that will be used in computing the desired confidence bounds is based on prior data indicating that 42/69 (61%) PTLD tissue samples were EBV+, while 27/69 (39%) were EBV−. Assuming this ratio will be the same in the 630 tissue samples, we will study in this aim, we base our computations on 384 EBV+ tissue samples and 246 EBV− samples. Table 2 contains the relevant 95% confidence bounds. It indicates, for example, that if we assume that a viral grouping is present in 20% of EBV+ and 20% of EBV− samples, then we can be 95% certain the true between-group difference in prevalence is at most 6%.

**TABLE 2.** 95% confidence bounds on the between-group difference in viral prevalence

Assumed prevalence	5%	10%	20%	30%	40%	50%
95% confidence bounds on difference	(–0.03 to 0.03)	(–0.05 to 0.05)	(–0.06 to 0.06)	(–0.07 to 0.07)	(–0.08 to 0.08)	(–0.08 to 0.08)

Results are calculated under the assumption that the prevalence is the same in EBV positive and EBV negative tissue.

Aim 3 does not have a power analysis presented; we do not currently know the different genome variants, and their frequencies, across all the EBV genes. The work we will do in this project will allow us to obtain this necessary information. We will then be able to assess associations of specific genome variants to likely pathogenicity and patient survival outcomes. As a preliminary example, of the three genes in our hypothesis that have a higher proportion of nonsynonymous variants, a characteristic 30 bp deletion in LMP1 was present in 7 of 33 EBV+ PTLDs, of whom 3/7 died within 5 y of PTLD diagnosis. With a larger sample size, we can associate this deletion with patient survival, in comparison with other LMP1 gene variants.

## CHARACTERISTICS OF OUR STUDY COHORT SO FAR

Our study commenced in summer 2019 and the COVID pandemic introduced significant restrictions on all clinical research activity in early 2020, thus slowing down our progress.

Despite these hurdles, as of this writing, we have accumulated 474 samples (360 FFPE, 95 plasma, 19 frozen tissue). Clinical data have been collected so far on 305 patients, of whom 42% were of pediatric age (under 18 y) at time of the qualifying organ transplant that was associated to the PTLD tissue collected. Demographic characteristics of this cohort are shown in Table 3. Early analyses that have been presented at major national and international meetings have focused on (a) IGH sequencing of repeat PTLD cases in the same patients; (b) association of end of treatment imaging results to subsequent patient survival; and (c) characterization of outcomes in early lesion cases. Full sequencing is ongoing.

**TABLE 3.** Demographic characteristics of our assembled PTLD cohort (n = 305 patients)

Variable	Category	N (%)
Transplant below age 1	Yes	26/305 (8.52%)
Pediatric patient (0–18 y)	Yes	129/305 (42.3%)
Gender	Male	178/305 (58.36%)
	Female	127/305 (41.64%)
Race	Asian	13/305 (4.26%)
	Other	20/305 (6.56%)
	Caucasian	234/305 (76.72%)
	Unknown	7/305 (2.3%)
	Black	28/305 (9.18%)
	Mixed	3/305 (0.98%)
Transplant organ	Heart	71/304 (23.28%)
	Hematopoietic stem cell	18/305 (5.9%)
	Lung	61/305 (20%)
	Kidney/Renal	74/305 (24.26%)
	Multi-organ Transplant	22/305 (7.21%)
	Liver	49/305 (16.07%)
	Intestine	9/305 (2.95%)
	Other	1/305 (0.33%)
Donor CMV status	Positive	64/146 (43.84%)
	Negative	58/146 (39.73%)
	Unknown	24/146 (16.44%)
Donor EBV status	Positive	56/143 (39.16%)
	Negative	13/143 (9.09%)
	Unknown	74/143 (51.75%)
Recipient CMV status	Positive	62/235 (26.38%)
	Negative	95/235 (40.43%)
	Unknown	78/235 (33.19%)
Recipient EBV status	Positive	51/231 (22.08%)
	Negative	71/231 (30.74%)
	Unknown	109/231 (47.19%)
Locations of PTLD	Lymph node	160/305 (52.46%)
	GI tract	92/305 (30.16%)
	Kidney	15/305 (4.92%)
	Liver	34/305 (11.15%)
	CNS	12/305 (3.93%)
	Disseminated	28/305 (9.18%)
	Bone marrow	10/305 (3.28%)
	Lung	64/305 (20.98%)
	Other	96/305 (31.48%)
	Unknown	6/305 (1.97%)
Was Rituximab given to treat PTLD?	Yes	211/295 (71.53%)
Did a second PTLD event occur after complete remission of the first PTLD event at any point in time during follow-up?	Yes	36/236 (15.25%)
Is the recipient alive?	No	210/305 (68.85%)
	Yes	88/305 (28.85%)
	Lost to follow up	3/305 (0.98%)
	Unknown	4/305 (1.31%)
Cause of death	PTLD related cause	114/209 (54.29%)
	Unknown	36/210 (17.14%)
	PTLD unrelated cause	59/210 (28.1%)
	Unknown	1/210 (0.48%)
Was systemic chemotherapy given to treat PTLD?	No chemotherapy used	114/305 (37.38%)
	New chemotherapy start	156/305 (51.15%)
	Unknown	33/305 (10.82%)
	Change to chemotherapy	2/305 (0.66%)
Years from transplant to initial PTLD diagnosis	Early PTLD (≤1 y)	100/305 (32.79%)
	Late PTLD (>1 y)	205/305 (67.21%)

CMV, Cytomegalovirus; CNS, Central nervous system; EBV, Epstein-Barr virus ; PTLDs, post-transplant lymphoproliferative disorders.

While we are collecting the peripheral blood EBV DNA loads, protocols may have changed considerably over that time. Changes in electronic medical records systems also limit our ability to capture some of the load data. We may have enough sample size to look at longitudinal changes in EBV load within the same patient.

## CLINICAL IMPLICATIONS

The potential clinical implications of the future findings of our study include (a) associations of EBV genetic variants to presentation severity, that may also allow for improved prognostication; (b) TTV loads may act as a simpler prognosticator; and (c) EBV genome variants that affect PTLD pathogenesis may also allow for future therapeutic targets.
